# Hydrophobia

**Published:** 1870-10

**Authors:** 


### HYDROPHOBIA.

Intense anger or hurt feelings can make a dog mad, hydrophobic, in an hour, and a bite during that state of feeling is death by hydrophobia. We have before stated that a man caught a dog, held it by the tail, and whipped it with a reed. The animal managed to bite him, and he died with hydrophobia; a few hours later, when the wife attempted to bring the dog from under the sofa, she was bitten, but suffered no harm. This occurred in Jersey City a year ago.

Within a month, in one of the Western States, a dog followed the family going on a pic-nic; they drove him back to the house, where the lame father had been left alone with his crutches; the dog came in and laid down, the wind blowing the door to; the dog seemed uneasy—began to whine; presently the hair stood on end, and he began to run around the room with great rapidity, and finally came to the bed where the helpless man lay; as the dog jumped up, with his fore paws on the side of the bed, the man gave him a tremendous stroke with his crutch. The dog retreated for a while, and then began to run around the room again. By this time the invalid began to look for the return of his wife and children, and concluded the moment they entered the door the dog would dash at them; this thought nerved him with a new energy: he rose in bed, on his knees, determined to strike at the dog every time he came around; in a short time, by successive strokes, the dog was disabled, and the heroic invalid beat him over the head with his crutch until the animal was a shapeless mass; in a short time after, the family returned. No doubt many a dog, not mad, but supposed to be, has been worried into madness by the hooting, stone-throwing mob at his heels.

Moral—Don't worry domestic animals. The bite of a mouse, the other day, which had been smeared with oil and set on fire, killed the two little children who did it.

The muzzling of dogs brings on hydrophobia, for it is a continual worry to them. From careful accounts kept, it has been found in parts of Germany that mad dogs were more numerous when the animals were muzzled, and less when the muzzling was discontinued by law.

It is perfectly certain that imagination, or other unnatural condition of the brain and nervous system, can originate hydrophobia in man.

The great practical point is, that as the poison of bites and stings is an acid, the strongest alkali known would be likely, by its immediate application, to nullify the poison; the alkali is spirits of hartshorn; when bitten then, four things should be done:

1. Preserve the dog's life; this may afterwards prove that he was not mad, and be an infinite relief to the person bitten.

2. Dabble a rag dipped in spirits of hartshorn on the bitten part for an hour.

3. Do all that is possible in the way of making light of it, or divert the mind from the subject, and never refer to it.

4. Administer the hartshorn internally ; dose, half a teaspoonful in a wine-glass of water, to a grown person ; half the amount in a tablespoon of water to children under twelve. Repeat daily under the direction of a physician for several days. For the bites of snakes, centipedes, scorpions, adders, &c., this method of internal treatment has been adopted by the governments of India, New South Wales, Victoria Tasmania, Queensland, South Australia, and to some in Egypt.

Eminent men claim that if the bite is instantly burned with a white-heated iron, three-fourths of the cases are saved. It ought to be known that hydrophobia is quite as common in winter as in summer.

